# Hepatoprotective Potential of Chestnut Bee Pollen on Carbon Tetrachloride-Induced Hepatic Damages in Rats

**DOI:** 10.1155/2013/461478

**Published:** 2013-10-22

**Authors:** Oktay Yıldız, Zehra Can, Özlem Saral, Esin Yuluğ, Ferhat Öztürk, Rezzan Aliyazıcıoğlu, Sinan Canpolat, Sevgi Kolaylı

**Affiliations:** ^1^Maçka Vocational School, Karadeniz Technical University, 61750 Trabzon, Turkey; ^2^Department of Chemistry, Faculty of Sciences, Karadeniz Technical University, 61080 Trabzon, Turkey; ^3^Department of Chemistry, Artvin Çoruh University, 08000 Artvin, Turkey; ^4^Department of Histology and Embryology, Faculty of Medicine, Karadeniz Technical University, Trabzon, Turkey; ^5^Department of Molecular Biology and Genetics, Faculty of Sciences, Canik Basari University, 55080 Samsun, Turkey; ^6^Faculty of Pharmacy, Karadeniz Technical University, 61080 Trabzon, Turkey; ^7^Department of Physiology, Faculty of Medicine, Karadeniz Technical University, Trabzon, Turkey

## Abstract

Bee pollen has been used as an apitherapy agent for several centuries to treat burns, wounds, gastrointestinal disorders, and various other diseases. The aim of our study was to investigate the hepatoprotective effects of chestnut bee pollen against carbon tetrachloride (CCI_4_)-induced liver damage. Total phenolic content, flavonoid, ferric reducing/antioxidant power, and DPPH radical activity measurements were used as antioxidant capacity determinants of the pollen. The study was conducted in rats as seven groups. Two different concentrations of chestnut bee pollens (200 and 400 mg/kg/day) were given orally and one group was administered with silibinin (50 mg/kg/day, i.p.) for seven days to the rats following the CCI_4_ treatment. The protective effect of the bee pollen was monitored by aspartate transaminase (AST) and alanine transaminase (AST) activities, histopathological imaging, and antioxidant parameters from the blood and liver samples of the rats. The results were compared with the silibinin-treated and untreated groups. We detected that CCI_4_ treatment induced liver damage and both the bee pollen and silibinin-treated groups reversed the damage; however, silibinin caused significant weight loss and mortality due, severe diarrhea in the rats. The chestnut pollen had showed 28.87 mg GAE/g DW of total phenolic substance, 8.07 mg QUE/g DW of total flavonoid, 92.71 mg Cyn-3-glu/kg DW of total anthocyanins, and 9 mg **β**-carotene/100 g DW of total carotenoid and substantial amount of antioxidant power according to FRAP and DPPH activity. The results demonstrated that the chestnut bee pollen protects the hepatocytes from the oxidative stress and promotes the healing of the liver damage induced by CCI_4_ toxicity. Our findings suggest that chestnut bee pollen can be used as a safe alternative to the silibinin in the treatment of liver injuries.

## 1. Introduction

Apitherapy has been used in folk medicine since the early ages of human beings, and in the recent years, its application in the treatment of burns, wounds, gastrointestinal disorders, ulcers, and carcinogenesis has been increasing. Bee pollen is one of the richest and purest natural foods that way ever discovered; the tremendous nutritional and medicinal value of the pollen has been used for centuries. Bee pollen is a perfectly balanced food and is rich in amino acids, proteins, hormones, enzymes, carbohydrates, minerals, fats, a considerable amount of vitamins, phenolic substances, phytochemicals, and significant quantities of antioxidant agents [1–3]. The chemical composition of the bee pollen depends on its botanical and geographical properties. Pollen contains about 1–5% total phenolic substances, which include different subtypes such as flavonoids, phenolic acids, anthocyanins, and tannins. They exhibit a wide range of biological activities including antioxidant, antimicrobial, anti-inflammatory, antiatherogenic, anticarcinogenic, and antithrombotic activities [1–4]. Phenolic compounds are considered to be beneficial for human health since they decrease the risk of degenerative diseases caused by oxidative stress. Many researchers have demonstrated that the phenolic compounds within the pollens inhibit the occurrence and development of numerous degenerative disorders [2]. 

Carbon tetrachloride (CCI_4_) is a hepatotoxic agent that enhances the formation of free radicals, which cause lipid peroxidation of cellular and organelle membranes [5], thus one of the most widely used toxic agents to induce liver diseases in animal models. CCI_4_ is metabolized by cytochrome p450 system to highly reactive trichloromethyl free radicals and reactive oxygen species, which initiate lipid peroxidation and necrosis. In addition, the toxic agent causes inflammatory response initiated by the activated hepatic macrophages, mainly Kupffer cells [6]. Many researchers have pointed out that CCI_4_ exposure could cause significant biochemical disorders such as fatty liver, hepatitis, and cirrhosis in laboratory animals [7, 8].

It is recognized that hepatocyte damage is one of the serious pathological disorders for human. To measure aspartate transaminase (AST) and alanine transaminase (ALT) activities in the serum or plasma is the simplest method to diagnose hepatocyte injuries [3, 9–11]. Oxidative stress was shown as one of the major causes of liver injury [11]. Silibinin or silybin is the active component of silymarin and commonly used for the treatment of various liver damages. Silymarin is obtained through the extraction of milk thistle seeds (*Silybum marianum*), which is rich in flavonoids, and used as a hepatoprotective drug for several decades. It has been shown that the bee pollen, which contains many phenolic substances, has similar effect as silibinin in terms of hepatoprotection [1, 2, 12]. For this purpose, many natural extracts and honey bee products, such as honey and pollen, were used to treat hepatic disorders in laboratory animals [3, 4, 9, 10]. 

In this study, we determined the therapeutic effects of chestnut bee pollen on the CCI_4_-induced liver damage in the rat model. We analyzed the chemical and antioxidant properties of the chestnut pollen and determined that pollen supplementation recovered the body weight, AST and ALT enzyme levels, malondialdehyde (MDA), and superoxide dismutase (SOD) levels as well as decreased the histological damage and apoptosis at the hepatocytes following the CCI_4_ treatment. We detected that both the bee pollen- and silibinin-treated groups reversed the CCI_4_-induced hepatic damage; however, silibinin caused significant weight loss and mortality due to severe diarrhea in the rats. Our results suggest that chestnut pollen can be used as a safe alternative for the treatment of liver damaging diseases.

## 2. Material and Methods

### 2.1. Reagents

CCI_4_, silibinin, gallic acid, quercetin, ethyl alcohol, methanol, Trolox (6-hydroxy-2,5,7,8-tetramethylchroman-2-carboxylic acid), 2,4,6-tripyridyl-s-triazine (TPTZ), Folin-Ciocalteu's phenol reagent, 2,2-diphenyl-1-picrylhydrazyl (DPPH), cyanidin-3-O-glucoside, TBA, 1,1,3,3-tetramethoxypropane, *β*-carotene, nitroblue tetrazolium, xanthine, and xanthine oxidase were purchased from Sigma Chemical Co. (St. Louis, MO, USA). AST and ALT diagnostic kits were also purchased from Sigma. Olive oil was obtained from KOMİLİ Sızma Company (İzmir, Turkey). 

### 2.2. Bee Pollen Samples

Pollen samples were obtained from the expert beekeepers of Zonguldak, Turkey (Western Black Sea area) in 2008 flowering season. The samples were dried at 40°C oven and palynological identification was done by Dr. Sibel Silici of Erciyes University, Turkey. Nine families of pollen pellets were found in the sample: Fabaceae (*Medicago* spp., *Trifolium* spp.), Fagaceae (*Castanea sativa* L.), Asteraceae (*Aster* spp., *Cirsium* spp., *Carduus* spp.), Apiaceae (*Apium* spp.), Caryophyllaceae (*Dianthus* spp.), Poaceae (*Zea may*), Rosaceae (*Malus* spp.), Myrtaceae (*Myrtus communis*), and Rhamnaceae (*Rhamnus cathartica*). Chestnut* sativa* was dominant (>45%) in the pollen mixture.

For the analysis of antioxidant potential of the pollen, the samples were prepared by mixing 1 g of dried powder of pollen sample with 10 mL methanol in a flask attached condenser, then sonicated in a sonicator apparatus (Elma Transsonic Digital, Germany). After 3 h sonication, the extract was used for antioxidant tests. Although we dissolved the pollen in sterile H_2_O to feed the animals by gavage, it has been shown that the antioxidant potential of the pollen is revealed with better percentage when it is extracted using methanol [13, 14].

Chemical analysis of the bee pollen was performed according to the method described at AOAC [15], and the values were calculated as per dried pollen weight (DW). In addition, the following potential antioxidant properties were measured according to the references mentioned: total phenolic content [16], total flavonoids [17], and total anthocyanins [18], total carotenoids [17], total antioxidant activity according to the ferric reducing antioxidant power (FRAP) [19] and to free radical scavenging activity of 2,2-diphenyl-1-picrylhydrazyl (DPPH) [20]. FRAP values were expressed as Trolox equivalent antioxidant power, and radical scavenging activity of DPPH was expressed as SC_50_, which represents the concentration of the extract (mg/mL) required to inhibit the 50% of the free radical scavenging activity. The lower SC_50_ value indicates the higher antioxidant activity.

### 2.3. Animals and Experimental Procedure

Forty-nine Sprague-Dawley rats were studied, which were 12 weeks old and of 250–300 g approximate weight. Animals were fed with standard rat feed and allowed to drink water. Animals were kept in temperature controlled (20–25°C) cages with 12 h dark and 12 h light cycles. *Food *was withdrawn for 12 h before the experiments. 

Animals were divided into seven groups (see Supplementary Table 1 in Supplementary Material available online at http://dx.doi.org/10.1155/2013/461478). The first group (G1–G3) was used as controls and other experimental groups. The control groups received 0.8 mL of saline (0.9% v/v) in water [21], olive oil (0.8% v/v) and ethanol (0.2% v/v) once per day for the entire period (7 days) by i.p. injection, respectively. The rats in G4 to G7 were administered i.p. with CCI_4_ dissolved in olive oil at a dose of 0.85 mL/kg/day body weight. G5 was fed with silibinin dissolved in ethanol at a dose of 50 mg/kg/day. G6 and G7 were fed with pollen at 200 mg/kg and 400 mg/kg once per day by gavage, respectively. In this study, dried pollen samples were dissolved in deionized water. This study was approved by the Animal Care and Use Committee from the Faculty of Medicine in Karadeniz Technical University (KTU).

### 2.4. Determination of Liver Enzyme Activities and Proteins

Plasma was obtained from the whole blood samples of the treated rats through centrifugation at 2000 ×g for 10 min and stored at −20°C until analysis. AST and ALT activities were measured by a Roche Diagnostics Modular Analyzer using the manufacturer's commercial kits according to the instructions (Roche Diagnostics GmbH, D-68298, and Mannheim, Germany). 

Reduction of nitroblue tetrazolium by xanthine-xanthine oxidase system was used to measure superoxide dismutase (SOD) activity in erythrocyte hemolysate of the rat blood samples. Formazan formation was examined at 560 nm using the spectrophotometer (Beckman-coulter, DU 530). The enzyme activity that causes 50% inhibition was regarded as one unit using bovine erythrocytes SOD as standard, and the results were read as U/g Hb [22].

MDA levels were measured with a colorimetric test with thiobarbituric acid (TBA) which is used to assess endogenous lipid [23]. Fresh tissue samples obtained from the treated rats were kept at −80°C until the analysis. Liver tissues were weighed and homogenized in ice-cold 1.15% KCl. The homogenate was centrifuged at 2000 ×g for 10 min. The breakdown product of 1,1,3,3-tetramethoxypropane was used as standard, and tissue MDA levels were calculated as nmol/mL plasma or g tissue. Total protein of the liver extracts was analyzed using Lowry et al. [24] method with bovine serum albumin as the standard. The values were achieved by interpolation on a calibration standard curve at 650 nm. 

### 2.5. Histological Preparation and Analysis

For histological analysis, liver tissue samples were fixed immediately in 10% buffered formaldehyde, dehydrated with ethanol series, cleaned with xylene, embedded in paraffin, and sectioned as 5 *μ*m. Tissue sections were stained with hematoxylin and eosin (H&E) then examined under a light microscope (Olympus BX-51; Olympus Optical Co, Ltd, Tokyo, Japan). All liver tissue slides were examined at high magnification and images were recorded by an independent histologist. Five high-power fields were selected by random sampling and the following criteria were followed in semiquantification of the liver injuries: hepatocyte degeneration, vascular congestion, sinusoidal dilatation, congestion in enlarged sinusoids, and fatty degeneration. Each specimen was marked using a scale of 0 to 3 (0 = none, 1: mild, 2: moderate, and 3: severe). The mean histologic score was calculated for each group.

### 2.6. TUNEL Analysis

For the detection of apoptotic cells within our groups (G1–G7), 4 *μ*m thick serial sections were prepared from the paraffin-embedded liver samples. For TUNEL analysis, *In situ* Cell Death Detection Kit (Roche, Mannheim, Germany) was used in accordance with the manufacturer's instructions to detect the fragmented DNA associated with apoptotic cells and clearly visible nuclear fragments or sharp and condensed chromatic masses or crescent in the nuclei. The stained sections were evaluated under a light microscope (Olympus BX51 microscope, Tokyo, Japan) at ×400 magnification. One hundred cells were counted per liver slide in five microscopic fields. The percentage of TUNEL-positive apoptotic cells was calculated and this represented the apoptotic index (AI). An independent histologist examined the stained specimens in a blinded fashion [11]. 

### 2.7. Statistical Analysis

The results were presented as mean values and standard deviations. Data and regression analyses were performed via Microsoft Office Excel 2003 (Microsoft Corporation, Redmond, WA). Data were tested using SPSS (version 9.0 for Windows 98, SPSS Inc.). Statistical analyses of the results were based on Kruskal-Wallis test and Pearson correlation analysis, which is a nonparametric test. The significance of the differences was statistically considered at the level of *P* < 0.05. 

## 3. Results and Discussion

This study investigated the hepatoprotective potential of aquatic extracts of chestnut bee pollen on CCI_4_-induced hepatic damages in rats. Before the treatments, some chemical and antioxidant properties of the pollen were investigated, which are summarized in Table 1. The main components of the pollen were protein, starch, fat, minerals, and water. The chestnut pollen has approximately 28.87 mg GAE/g of total phenolic compounds (TPC), 8.07 mg QUE/g of total flavonoids, 92.71 mg Cyn-3-glu/kg of total anthocyanins, and 29 mg *β*-carotene/100 g of total carotenoids. These compounds are well established for their responsibility of the antioxidant activities of the natural honeybee products [1, 25]. The chestnut pollen also demonstrated substantial antioxidant activity, which is measured through ferric reducing antioxidant power (FRAP) and DPPH radical scavenging activity (Table 1). Although there are other honeybee products with higher bioactivity, such as royal jelly and propolis, chestnut pollen was used in this study due to its water solubility and relatively higher antioxidant capacity. Cheng et al. [26] recently studied the antioxidant and hepatoprotective effects of *Schisandra chinensis* pollen extract (SCPE) on CCl_4_-induced acute liver damage in mice and found that SCPE had strong antioxidant activities and significant protective effect against the acute hepatotoxicity induced by CCl_4_, which was also supported by the evaluation of liver histopathology in mice. Furthermore, in another recent study, Tohamy et al. [27] studied the antioxidant capacities of the water extracts of the Egyptian bee pollen (WEBP) on cisplatin (CDDP)-induced damages in multiple organs and found that WEBP was more potent in the recovery of hepatic and testicle damages.

The phenolic compounds of the pollens vary depending on the geographic characteristics and the flora of the region [1]. Recently, Ulusoy and Kolayli [13] studied the Anzer pollen from the Black Sea Region of Turkey and found that TPC of Anzer pollen ranged from 44.07 to 124.10 mg GAE/g, which is higher than the chestnut pollen. The TPC of the bee pollen was also studied by Mărghitaş et al. [28] using twelve types of pollen collected from Romania. In this study, the TPC ranged from 6.40 to 16.4 mg GAE/g dry matter of pollen, and Salix type of pollen showed the highest content of total phenolic and antioxidant activity. In another study, the phenolic content (polyphenolics, flavonols, flavones, and flavonones) and the antioxidant activity in six varieties of bee pollen from Sonoran Desert were studied [14]. They found that the phenolic content ranged between 15.91 and 34.85 mg GAE/g pollen, and a great variation was observed among the pollen samples. There was no previous study found about the phenolic content and antioxidant activity of chestnut pollen to make comparison with our results; however, there are a few studies about chestnut honey that contains crucial amounts of phenolic substances that are related to biological activities, such as antioxidant, antimicrobial, and anti-inflammatory features [29, 30]. 

We measured the initial weight of the 49 rats that we studied in 7 groups (G1–G7) and compared their weights at the end of the 7th day (Table 2; Supplementary Table 1). While the rats in the first two control groups of physiological saline (G2) and olive oil (G3) increased in weight (0.7%), the ethanol group (G3) decreased in weight (5.5%). Meanwhile, weight loss was detected in all of the CCl_4_-injected groups (G4–G7); the untreated CCl_4_ control group (G4) and silibinin-treated group (G5) showed the highest weight loss with −9.5% and −8.2%, respectively (Table 2). In addition, the significant weight loss in the silibinin group was due to severe diarrhea and caused mortality of 1 rat (out of 7) within the group (G5). Meanwhile, the weight loss was the least amount in the pollen-treated groups (G6-G7). The more the amount of pollen fed, the less the weight loss in the rats. This suggests that the bee pollen acted as an efficient nutrition supplement in the CCl_4_-exposed rats, without any substantial side effect and mortality. 

The activities of plasma AST and ALT enzymes were significantly elevated in rats treated with CCI_4_ due to toxic effect (Table 3). Although the enzyme activities were not changed in the control groups (G1–G3), a significant increase in the CCI_4_-treated groups (G4–G7) was observed. AST and ALT enzyme activities were markedly increased in all the CCI_4_ administered groups, especially 4-5 times increased in the G4 (CCI_4_-only group) than the control groups (G1–G3). In case of the intake of silibinin and the bee pollen were reduced both enzyme activities. ALT and AST enzymes are specific to liver damage; they are used routinely for the determination of liver injuries [31]. When the bee pollen was administered at higher doses of 400 mg/kg/day, AST and ALT levels were significantly decreased. In the present study, the silibinin-treated (50 mg/kg/day) rats (G5) showed better improvement than the low-dose pollen-treated (200 mg/kg/day) group (G6); however, as a critical side effect, severe diarrhea and weight loss were detected in the silibinin-treated group. Furthermore, the improvement of AST and ALT levels within the pollen-treated groups (G6 and G7) correlated with the concentration of the pollen given to the rats. 

Among the apitherapy studies related to the prevention and protection of liver damage and toxicity, Türkez et al. [10] showed that propolis extracts inhibited the liver damage induced by AlCl_3_ treatment in the mouse model. Similarly, Liu et al. (2012) [9] reported that quercetin, an antioxidant substance found in honeybee products, prevented liver injury from lead-induced oxidation. Kolankaya et al. [32] showed that the propolis of* Castanea sativa* showed a protective effect against alcohol-induced liver damage. El-Denshary et al. [33] indicated that dietary honey consumption reduced the hepatotoxicity against CCI_4_-induced liver damage. Recently, there are some animal model studies that showed that many honeybee products exert curative potential for the protection of hepatocytes from oxidative damages [3, 10, 31, 32].

Malondialdehyde (MDA), a lipid peroxidation product, was measured in plasma, erythrocyte, and liver tissues of the treated animals to analyze the damage caused by oxidative stress induced via CCI_4_ treatment. The amount of MDA in liver tissue serves as an indicator of lipid peroxidation, which is a well-known occurrence in the liver injury due to generation of reactive species [34]. Within the liver tissues, there was not a significant alteration detected in the MDA levels of the control groups (G1–G3), whereas the MDA level was significantly increased in the sick group (G4), which was almost 5 times more than G1–G3. A substantial decrease was observed in the MDA levels of the rats, which were exposed to silibinin and pollen groups (G5–G7) (*P* < 0.05). As a result of CCI_4_ treatment, MDA levels were increased in the plasma, erythrocytes, and liver, which have demonstrated that CCI_4_ caused lipid peroxidation. Liver plays a major role in the metabolism of xenobiotic; thus, it is vulnerable to many compounds, which are either toxic as themselves or produce metabolites that can cause liver damage [9, 34]. The efficiency of silibinin against oxidative stress in liver was reported in the previous studies [12]. Our results clearly showed that both the silibinin and the bee pollen are effective in the prevention of lipid peroxidation, as well as oxidative damage, which was induced by CCI_4_. However silibinin caused mortality due to severe diarrhea in our rat model.

Superoxide dismutase (SOD) is an important antioxidant enzyme that protects the organism from the harmful effects of superoxide radicals formed as a result of oxidative stress [4]. In our study, there was no significant difference detected in SOD activities in plasma, liver, and erythrocyte among the control groups (G1–G3), whereas SOD activity was decreased in the CCI_4_-exposed groups. The lowest SOD activity was detected in the rats exposed to CCI_4_ (G4), and the activity slightly increased in the groups that were silibinin- and pollen-administered groups (G5–G7). SOD is physiologically synthesized in the liver cells similar to other antioxidant enzymes; thus, the protein synthesis is negatively affected as a result of the hepatocellular injury induced by CCI_4_. However, SOD activity was increased in pollen- and silibinin-treated groups, possibly, owing to the healing of hepatocellular damage. Similar to our results, it has been shown that SOD activity was decreased notably in the mice treated with trichlorfon, which is an organopesticide, but significantly increased in the groups which were given pine honey [3]. The results of our present study are in agreement with the previous studies in terms of alterations in oxidative stress markers in response to apitherapy [3, 31].

**Figure 1:**
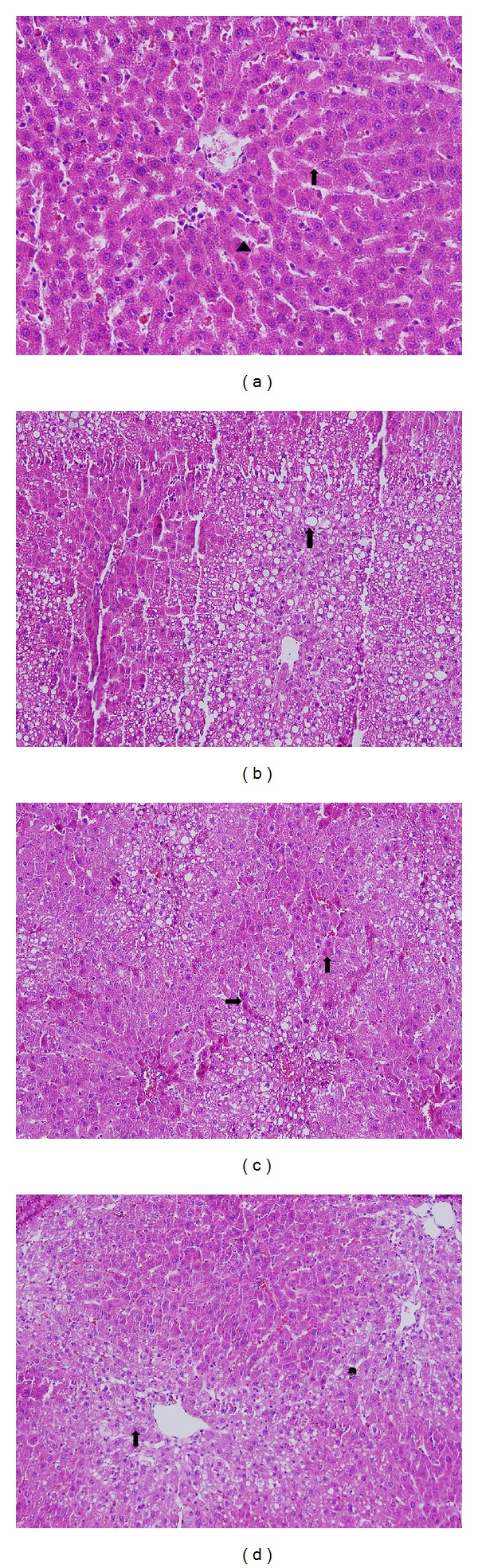
Histopathological analysis of treated and untreated liver sections. (a) Normal hepatocytes (↑) and sinusoids (▲) in the control group, untreated CCI_4_ (H&E ×100). (b) Destroyed group with CCI_4_ (G4), increased fatty degeneration (↑). (c) Pollen-treated group (G6) with 200 mg/kg, decreased fatty degeneration and regeneration in hepatocytes (↑). (d) Pollen-treated group (G7) with 400 mg/kg pollen, fatty degeneration markedly decreased (↑).

Microscopic examination showed liver parenchyma and sinusoids of the hepatocytes were healthy in the control groups (G1–G3) (Figure 1(a)). Meanwhile, obvious damages were observed in the liver sections of the CCI_4_-exposed group. Common degeneration and noticeable fatty vacuolization were observed in the hepatocytes from the sick group (G4). There was vascular congestion in the sinusoids (Figure 1(b)). A slight degree of vacuolar degeneration and fatty vacuolization were observed in the hepatocytes of G5 (silibinin), G6, and G7 (pollen-administered groups); however, the level of vacuolar degeneration of these groups was less than that of G4. And also, histological improvement of G7 (400 mg pollen/kg/day) was better than that of G6 (200 mg pollen/kg/day) (Figures 1(c) and 1(d)). The improvement was significantly relevant with the bee pollen amount given to the animals. Histological damage scoring and statistical evaluation of groups were shown in Table 3.

**Figure 2:**
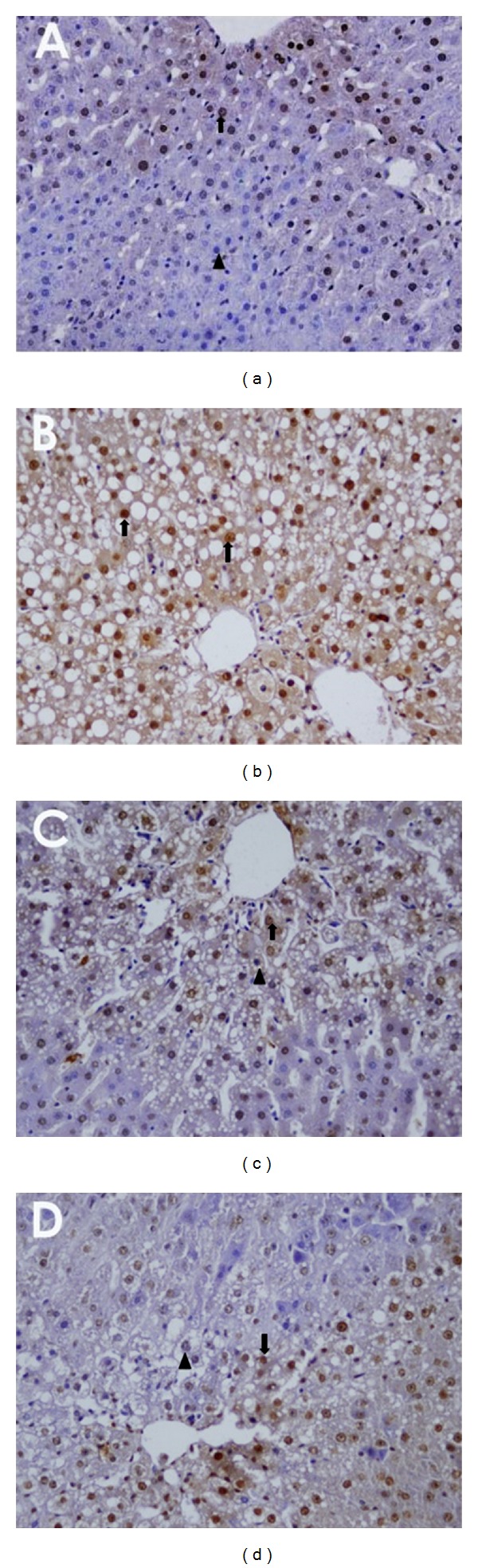
Results on apoptosis analysis (TUNEL) of CCI_4_-induced apoptosis in primary rat hepatocytes, apoptotic hepatocytes (↑), and normal hepatocytes (▲) (×400). (a) Control group untreated with CCI_4_ (G1), brown labeled apoptotic hepatocytes (↑), and blue labeled normal hepatocytes (▲). (b) Destroyed with CCI_4_ (G4), apoptotic hepatocytes (↑). (c) Pollen-treated group (G6) with 200 mg/Kg. (d) Pollen-treated group (G7) with 400 mg/Kg.

Hepatocyte apoptosis was analyzed using terminal deoxynucleotidyl transferase-mediated deoxyuridine triphosphate nick end-labeling (TUNEL) assay. The number of apoptotic nuclei was much higher in sick group (G4) than that of the control groups (Figures 2(a) and 2(b)); however, the number of apoptotic nuclei of G5–G7 (silibinin- and pollen-administered groups) was much reduced compared to the sick group (G4) (Figures 2(c) and 2(d)). The apoptosis values in the liver and the results of the statistical analysis are shown in Table 3. Histopathological damage scoring and TUNEL analysis demonstrated that tissue damage in the liver was higher in the groups exposed to CCI_4_ and found that the tissue damage was significantly reduced in the groups treated with pollen following CCI_4_ exposure. The apoptosis index (AI) was significantly increased in the group that was exposed to CCI_4_ without any treatment (G4) compared to the treatment groups (G5–G7) (Table 3). However, the AIs of these treatment groups were at least five times more than the control groups, which were not exposed to CCl_4_ (G1–G3). This suggests that the initial exposure of the cells to CCI_4_ massively increased the apoptosis of the hepatocytes; thus, the treatments of the rats with either silibinin or pollen could not completely reverse the devastating impact of CCI_4._

Experimental liver damage model is mostly achieved by exposing rats to CCI_4_. It has been shown that lipid peroxidation increases and free oxygen radicals are released in the rats exposed to CCI_4_ [7, 8]. Free oxygen radicals are known to be the mediators of tissue damage. Reactive oxygen radicals cause oxidative damage to cellular macromolecules such as nucleic acids, proteins, and lipids, which eventually lead to apoptosis. Free oxygen radicals cause lipid peroxidation, several metabolic disorders, and functional abnormalities through inducing membrane and DNA damages [35]. Lipid peroxidation is believed to be an important cause of destruction and damage to the cell membranes, which leads to changes in membrane fluidity and permeability. Moreover, lipid peroxidation also enhances the rate of protein degradation, which initiates the eventual lysis of the cells [11]. Our results indicated that the water pollen extracts inhibited the CCI_4_-induced apoptotic cell death and hepatotoxicity. It has been reported that free oxygen radicals play important roles in CCI_4_-induced cell injury [7]. When the amount of free oxygen radicals in the cell are increased, they overpower the defense systems and cause oxidative stress or cell injury, leading to development of various diseases. Thus, CCI_4_-induced apoptosis is related to oxidative stress in hepatocytes and intracellular antioxidants may protect hepatocytes against cell apoptosis induced by CCI_4_ [9].

## 4. Conclusion

 CCI_4_ is a hepatotoxic agent that enhances the formation of free radicals, which cause lipid peroxidation of cellular and organ damages. The chestnut bee pollen contains substantial nutrients and possesses many phenolic compounds, which are the factors of high antioxidant properties. Our study clearly shows that the chestnut bee pollen exerts highly beneficial biological activities in the protection of hepatocytes from oxidative stress and toxicity induced by CCI_4_ exposure. Therefore, we conclude that the chestnut bee pollen could be safely included in the daily human diet as a food additive, which will enhance the inhibition of the oxidative stress. Chestnut bee pollen could be used as a suitable alternative to silibinin in the treatment of hepatocellular pathologies.

## Supplementary Material

Supplementary Table 1: Palynological identification of pollen sources within the pollen samples used in the study
Supplementary Table 2: Experimental and control groups used in this study

## Figures and Tables

**Table:** (a)

Chemical properties				
Moistures %	Ash %	Protein %	Starch %	Fat %
14.42 ± 0.42	2.23 ± 0.21	23.67 ± 2.08	6.83 ± 6.83	5.87 ± 2.39

**Table:** (b)

Antioxidant properties					
Total phenolic content (mg GAE/g DW)	Total flavonoid (mg QUE/g DW)	Total anthocyanins (mg Cyn-3-glu/kg DW)	Total carotenoid (mg *β*-carotene/100 g DW)	FRAP (mM Trolox/g DW)	DPPH SC_50_ (mg/mL)
28.87 ± 2.48	8.07 ± 0.83	92.71 ± 25.21	29 ± 9.0	82.31 ± 2.41	0.62 ± 0.10

**Table 2:** Experimental results of transaminase enzymes and antioxidant parameters in the plasma (*n* = 7). Data were given as mean ± SD.

Groups	Weight gain %^(1)^	Plasma				Erythrocytes		Liver	
		AST	ALT	MDA	SOD	MDA	SOD	MDA	SOD
		U/L	U/L	nmol/mL plasma	U/mg protein	nM/g Hb	U/g Hb	nmol/g tissue	U/g liver
G1 (control)	+0.7 (±1.07)^b^	171.57 ± 21.74^a^	85.86 ± 14.31^a^	0.50 ± 0.02^a^	729.30 ± 30^c^	0.55 ± 0.3^a^	62.93 ± 31.5^bc^	4.85 ± 0.2^a^	540.10 ± 10.3^c^
G2 (control)	+0.7 (±1.07)^b^	207.14 ± 20.59^a^	86.57 ± 8.90^a^	0.51 ± 0.01^a^	750.10 ± 25^c^	0.53 ± 0.3^a^	80.93 ± 68.1^c^	5.82 ± 0.4^a^	520.20 ± 19.2^c^
G3 (control)	−5.4 (±0.946)^a^	145.50 ± 21.45^a^	44.50 ± 11.47^a^	0.53 ± 0.03^ab^	700.65 ± 45^c^	0.55 ± 0.1^a^	46.12 ± 19.5^ab^	5.95 ± 0.5^a^	515.15 ± 23.1^c^
G4 (CCI_4_)	−9.5 (±0.905)^a^	892.57 ± 29.11^d^	474.86 ± 134.76^d^	0.57 ± 0.08^b^	260.25 ± 15^a^	0.96 ± 1.3^a^	15.42 ± 21.7^a^	22.62 ± 2.7^e^	260.50 ± 42.1^a^
G5 (silibinin)	−8.2 (±0.918)^a^	518.80 ± 122^b^	218.80 ± 73.25^b^	0.53 ± 0.02^ab^	305.90 ± 40^b^	0.67 ± 0.3^a^	15.26 ± 14.1^a^	8.30 ± 0.8^b^	290.60 ± 23.2^b^
G6 (low pollen)	−6.7 (±0.933)^a^	690.83 ± 149^c^	383.40 ± 93.02^c^	0.53 ± 0.03^ab^	265.30 ± 30^a^	0.49 ± 0.3^a^	14.74 ± 6.6^a^	14.82 ± 1.5^d^	270.20 ± 12.3^bc^
G7 (high pollen)	−1.6 (±0.984)^ab^	464.0 ± 69.02^b^	277.00 ± 49.62^b^	0.52 ± 0.01^a^	270.25 ± 20^ab^	0.59 ± 0.5^a^	17.72 ± 8.7^a^	10.94 ± 0.9^c^	310.10 ± 14.4^b^

^(1)^Body weight (B.W.) changes during the experiments (mean ± SD); a positive (+) value shows an increase of the body of the rat whereas a negative value (−) shows decrease of the body of the rat.

^a^Values are significantly different from G4, G5, G6, and G7 (*P* < 0.05).

^b^Values are significantly different from those of controls (G1–G3) (*P* < 0.05).

^c^Values are significantly different from those of silibinin (G5) (*P* < 0.05).

^d^Values are significantly different from G7 (400 mg pollen/kg B.W.) (*P* < 0.05).

**Table 3:** Histologic analysis and scoring of liver sections of the treated and untreated rats.

Groups	Hepatocyte degeneration	Vascular congestion	Sinusoidal dilatation	Congestion in enlarged sinusoids	Fatty degeneration	Apoptosis index (AI)
G1 (control)	0.43 ± 0.535^a^	0.57 ± 0.535^ab^	0.29 ± 0.488^a^	0.29 ± 0.488^a^	0.0 ± 0.0^a^	3.57 ± 2.15^a^
G2 (control)	0.43 ± 0.535^a^	0.71 ± 0.488^abc^	0.86 ± 0.378^ab^	0.29 ± 0.488^a^	0.0 ± 0.0^a^	4.71 ± 1.70^a^
G3 (control)	0.29 ± 0.488^a^	0.43 ± 0.535^a^	0.71 ± 0.488^ab^	0.29 ± 0.488^a^	0.0 ± 0.0^a^	3.86 ± 1.68^a^
G4 (CCI_4_)	2.71 ± 0.488^c^	0.71 ± 0.488^abc^	1.57 ± 0.535^c^	0.86 ± 0.378^b^	3.00 ± 0.0^c^	35.57 ± 6.35^d^
G5 (silibinin)	2.00 ± 0.577^b^	1.14 ± 0.69^bcd^	1.29 ± 0.756^bc^	0.71 ± 0.488^ab^	1.86 ± 0.69^b^	20.0 ± 3.16^c^
G6 (low pollen)	2.14 ± 0.378^b^	1.43 ± 0.535^d^	1.29 ± 0.756^bc^	0.86 ± 0.378^b^	2.14 ± 0.69^b^	21.57 ± 4.04^c^
G7 (high pollen)	1.71 ± 0.488^b^	1.29 ± 0.488^cd^	1.29 ± 0.488^bc^	0.71 ± 0.488^ab^	1.71 ± 0.756^b^	15.86 ± 3.29^b^

The histological score was calculated using a scale from 0 to 3, 0: none, 1: mild, 2: moderate, and 3: severe.

^a^Values are significantly different from G4, G5, G6, and G7 (*P* < 0.05).

^b^Values are significantly different from those of controls (G1–G3) (*P* < 0.05).

^c^Values are significantly different from those of silibinin (G5) (*P* < 0.05).

^d^Values are significantly different from G7 (400 mg pollen/kg B.W.) (*P* < 0.05).
