# Supplementary material for: Hepatoprotective Potential of Chestnut Bee Pollen on Carbon Tetrachloride-Induced Hepatic Damages in Rats
Source: Evid Based Complement Alternat Med. 2013 Oct 22;2013:461478. doi: 10.1155/2013/461478 (PMC3819792; doi:10.1155/2013/461478)
Supplement: Supplementary file 1 — Supplementary Table 1: Palynological identification of pollen sources within the pollen samples used in the study Supplementary Table 2: Experimental and control groups used in this study [file 461478.f1.pdf]

**Supplementary Table 1.** Palynological identification of pollen sources within the pollen samples used in the study

| **Species** | **Percentage (%)** |
|---|---|
| Fabaceae (Medicago spp., Trifolium spp) | |
| Fagaceae (Castania sativa L.) | 45% |
| Asteraceae (Aster spp., Cirsium spp., Carduus spp) | |
| Apiaceae (Apium spp.) | |
| Caryophyllaceae (Dianthus spp.) | |
| Poaceae (Zea may) | |
| Rosaceae (Malus spp.) | |
| Myrtaceae (Myrtus communis) | |
| Rhamnaceae (Rhamnus cathartica) | |

**Supplementary Table 2.** Experimental and control groups used in this study

| **Groups** | **Treatment** |
|---|---|
| G1 *(Control)* | 0.9% NaCl (i.p.) |
| G2 *(Control)* | 0,8 ml/kg Olive oil (i.p.) |
| G3 *(Control)* | 0,8 ml/kg Ethanol (i.p.) |
| G4 *($CCI_4$-only)* | 0,8 ml/kg $CCI_4$ in olive oil (i.p.) only |
| G5 *(Silibinin)* | 0,8 ml/kg $CCI_4$ in olive oil (i.p.) + Silibinin (50 mg/kg/day) gavage |
| G6 *(Low Pollen )* | 0,8 ml/kg $CCI_4$ in olive oil (i.p.) + Pollen (200 mg/kg/day) gavage |
| G7 *(High Pollen)* | 0,8 ml/kg $CCI_4$ in olive oil (i.p.) + Pollen (400 mg/kg/day) gavage |

i.p.: Intraperitoneal
